# Contraceptive utilization and associated factors among polygamous and monogamous women in Worebabo Woreda, South Wollo Zone, Ethiopia: a comparative cross sectional study

**DOI:** 10.1186/s12905-023-02180-y

**Published:** 2023-01-30

**Authors:** Eueail Teferi Asrese, Yonas Fissha Adem

**Affiliations:** Department of Public Health, Dessie College of Health Sciences, P.O.Box: 1212, Dessie, Ethiopia

**Keywords:** Modern contraceptive utilization, Marital type, Associated factors

## Abstract

**Background:**

In Ethiopia high population growth and unintended pregnancies are posing pressures where the economy is incapable of holding overpopulation. Despite this problem, utilization of modern contraception is low in rural areas of the country, especially in the areas where polygamy is common. Therefore, this study was conducted to assess contraceptive utilization and associated factors among polygamous and monogamous women in, Ethiopia.

**Method:**

A community-based comparative cross-sectional and phenomenological study design was employed from July 1 to September 30, 2021, on the total sample size of 774 selected married women of the reproductive age group by using a multistage sampling method and a purposive sampling method were used for the qualitative part of the study. A pre-tested interview with a structured questionnaire was used to collect data and key informants were interviewed using semi-structured questionnaire. Associated factors were analyzed by using bivariable and multivariable binary logistic regression models. The odds ratio, with a 95% confidence level, was used to declare a statistically significant association.

**Result:**

A total of 703 married women of the reproductive age groups were interviewed, yielding a 90.89% response rate; among these married women, 352 and 351 were in monogamous and polygamous relationships. The proportion of women who use modern contraceptives was 161 (45.7%) in monogamous relationship, and 151 (43.0%) in polygamous relationships. Overall, utilization of modern contraceptives was significantly associated with educational status (AOR = 2.143, CI:1.428–3.216), religion (AOR = 1.704, CI: 1.144—2.539), undesired fertility (AOR = 3.17,CI:1.939–5.183), who decides on the number of children (AOR = 3.054, CI:1.93–4.832), getting clear information by Health care provider (AOR = 4.624, CI:3.132–6.828), family pressure (AOR = 1.855, CI:1.351–2.75), fear of social stigma (AOR = 2.482, CI:1.666–3.699), and accepts myths about contraceptives (AOR = 1.878, CI:1.278–2.761).

**Conclusion:**

This study identified that utilization of modern contraception was low in the study area. The district health office and concerned stakeholders should implement interventions that scale up contraceptive use, need family involvement in decision making, addressing myths around contraceptives, helping women to get education, and training of health care providers.

## Background

High fertility remains a public health problem, and the intention to reduce fertility is a global phenomenon [1]. Contraception also known as family planning is defined according to the World Health Organization, as a process whereby individuals couples decide voluntarily, free from coercion, desired or intended number of children, timing and spacing of their birth [2].

Contraception, according to the global community, helps to prevent an estimated 2.7 million infant deaths, the loss of 60 million healthy lives each year, and has the potential to reduce poverty and hunger, particularly in developing countries [3].

Compared to developed countries, modern family planning utilization is low in developing countries. In developing countries in 2017, it is estimated that there are 1.6 billion women of reproductive age (15–49) group, of these women 885 million want to avoid pregnancy, but only 671 million of them are using modern contraceptives [4]. Regionally, for married women, unmet need in sub-Saharan Africa is approximately 24%, twice the level of 12% in Latin America and the Caribbean [5]. several studies reported that the utilization of modern contraceptive service is determined by demographic and Economic characteristics, [6, 7] reproductive characteristics, personal and interpersonal factors, [8, 9] the supply-side factors, [10] access barriers, [11] and socio-cultural factors that are represented in the societal norms and practices [12, 13].

Currently, it is more evident that fertility preference is affected by the socio-cultural perspectives of a society. In developing countries, where socio-cultural identities are more deeply rooted, these factors have a great effect on the utilization of modern contraceptive methods [14]. Among these factors, one is the type of marriage especially polygamous union. In most African countries man having more than one wife is acceptable with a lot of attachment to children and wealth, bearing many children would mean security for the mother [15]. The prevalence and effect of polygamous relationships may have serious reproductive and health consequences for women [16].

In Ethiopia, the prevalence of polygamy was 11% in 2016 [17]. Even though government family law restricts polygamy, it is one of the socio-cultural disadvantages that women have to face. In Ethiopia studies show that women in polygamous marriage tend to have two times lower utilization of contraceptives than in monogamous marriage [18]. These is due to the fact that women in polygamous union are more likely to be older, have more children, less educated, live in a poorer household, reside in a rural area, competition among co-wives, reduced perception of the cost of children, lowered communication with spouse and early marriage and being young which all are associated with low contraceptive utilization [19]. The key factor driving the difference in women's fertility among polygamous women in rural Ethiopia appears to be marital rank [20]. But evidences on the predictors of modern contraceptive method utilization are scarce and not well known in the context of polygamous community in the study area. Therefore, this study was conducted to assess contraceptive utilization and associated factors among polygamous and monogamous women in Worebabo Woreda. So that the study findings will contribute to the development of context specific strategies and family planning programs in polygamous communities.

## Methods and material

### Study design, setting and period

A community-based comparative cross-sectional and phenomenological study was carried out in Worebabo Woreda, which is located in Amhara regional state's South Wollo zone. The administrative center of Werebabu is Bistma, and it has a total of 24 kebeles. According to the Woreda administrative health office report the total population of the Woreda in 2019 was 123,434. The study was conducted from July 1 to September 30, 2021.

### Population

#### Source population

The source population was all married reproductive-age women living in Worebabo Woreda.

#### Study population

The study population was all married reproductive age women in selected kebeles during data collection period.

### Inclusion and exclusion criteria

#### Inclusion criteria

All women who were in married reproductive age group (15–49).

#### Exclusion criteria

Women who have a history of hestroctomy and who were critically ill during the data collection period.

Women who were pregnant during the data collection period.

### Sample size determination

"The sample size for the quantitative study is calculated using the double population proportion formula, yielding 774 married reproductive-age women (each of polygamy and monogamy family sample sizes is 387), based on the assumptions of a 24.4% and 14.0% modern contraceptive utilization rate by monogamous and polygamous married women respectively, [3] 5% margin of error, a 95% confidence level, and a 10% non-response rate.

For the qualitative study, 15 study participants who were married reproductive-age women in each monogamous and polygamous relationship were used.

### Sampling procedure

For the quantitative part, a multi-stage sampling method was used to select study participants. From the total of twenty four kebeles, fifteen kebeles are selected using the lottery method. Pre-survey was conducted in selected kebeles to identify the number of married women in monogamous and polygamous relationships and was used as a sampling frame. The total sample size was allocated proportionally to each selected kebele by using the sampling frame from the survey result, and participants were selected by using a simple random sampling method. The data collector encountered more than one married woman in polygamous relationships at one house and chose one participant using a simple random sampling method.

For qualitative data, a purposive sampling method was used to select candidates in both polygamous and monogamous unions.

### Study variable

The dependent variable was the utilization of modern contraceptive methods, and the independent variables of the study were socio-demographic characteristics (women’s age, age of marriage, educational status, residence of living, duration after marriage, and wealth status), reproductive history of the women (number of previous pregnancies, number of previous deliveries, number of abortions, number of neonatal loss, number of still births, and fertility desire), knowledge about modern contraceptives, and socio-cultural perspectives (perceived acceptance by religious leaders, fear of social stigma, myths about contraceptives, family pressure and perceived misconceptions and fears of side effects of contraceptives).

### Operational definitions

*Modern contraceptive utilization*. The main outcome variable (dependent variable), which is dichotomized into modern contraceptive users and non-users [21].

*Modern contraceptive users*. Those women or whose husbands are using one of the modern contraceptive methods (oral contraceptive pill, injectable, implants, IUDs, condom, sterilization) during the data collection time [21].

*Modern contraception non-users*. Those women who or whose husband is not using a modern contraceptive method during the data collection time [21].

*Unmet need for contraception*. Broadly defined as those women who want to delay or stop childbearing but are not using contraception.

*Misconceptions’ and fears of side effects of contraceptives*. This variables were measured using the “yes” and “no” answers. The variables were collected by the use of a questionnaire and interviews [22].

*Monogamy.* Refers to unions in which there is one man and one wife. It was measured by “Yes” or “No” answers. The variables were collected by using the questionnaire and interview [18].

*Polygamy.* Refers to unions in which there is one man and more than one wife. It was measured by “yes” or “no” answers. The variable was collected by the use of questionnaire and an interview [18].

*Knowledge of family planning.* Refers to respondents’ previous knowledge regarding modern contraceptive methods. This was measured with the help of 8 knowledge related questions by using “yes” or “No” questions and 1pt for yes and 0 pt for no answers then based on cut off point (> 4) respondent classified as having a good knowledge otherwise poor Knowledge [23].

*Atittude about family planning.* Refers to respondents’ views and opinions towards the contraceptive methods. This was measured by with the help of different attitude-related questions.

## Data collection tools and quality control

For the quantitative study, the data was collected using structured interview administered questionnaires which adopted from different literatures [3, 21, 24, 25]. The questionnaires were translated into Amharic and back to English to ensure consistency. The data was collected by eight health extension workers, which were selected from other unselected kebeles and supervised by two BSc health professionals. Its quality was controlled by designing proper data collection tools, pre-testing, and continuous supervision and before actual data collection, training was provided to health extension worker data collectors for two days on the data collection techniques to familiarize data collectors with the tool.

For the qualitative part using a semi-structured interview guide as a tool in-depth interview was conducted on key informants to explore the experience of married women on the effect of socio cultural factors toward utilization of modern contraception. The guide was constructed from different literature [21, 26]. Increase the trust worthiness credibility was insured by approaching each study participant’s friendly, ensuring privacy and confidentiality before interview.

## Data analysis

Data were entered in Epi data 3.1 and it was checked, cleaned, and edited before analysis, and it was exported to SPSS version 25 for analysis. Frequency distributions and cross tabulations were used to check for missed values and outliers during the analysis. Descriptive statistics were computed as frequency, percentage, and results were displayed using tables and graphs. The relationship between the independent variable and the outcome variable was determined using a multivariable binary logistic regression model. Model fitness was checked by Hosmer and lemon show test, and multicollinearity was checked by VIF. The first bivariable analysis was made for each independent variable to the outcome variable, and those variables resulting *P*-value less than 0.2 were entered into the multivariable binary logistic regression model. In the final model, those variables with a *P*-value less than 0.05 were considered as statistically significant, and they were presented by odds ratio (OR), with a 95% confidence level (CI) to show the strength and direction of the association.

The qualitative data, which was obtained from participants conversations, was audio-taped, transcribed, translated and coded. The qualitative data was analyzed using thematic analysis. The investigator was read the collected data repeatedly, and codes it. The coded data categorized and then grouped in theme as per the objective of the study then it was displayed and reduced. Finally, the reduced data was interpreted.

## Result

A total of 703 married women of reproductive age were interviewed, yielding a 90.89% response rate; 352 of these women are in monogamous relationships, while the remaining 351 were in polygamous relationships.

### Socio-demographic character

For monogamy and polygamy, the mean age of respondents with standard deviation were 32.77 (± 7.68) and 37.65 (± 6.59), respectively. Concerning the educational status of the respondents, 44.3% and 51.6% of monogamous and polygamous women didn’t have any formal education, respectively, and the result shows a statistically significant difference at (X2 = 13.88, *P*-value = 0.002) between the two groups. With regard to respondent occupation, 47.4% and 53.0% Monogamous and polygamous women are farmers, respectively. Religion type 64.2% of monogamous women and 70.9% of Polygamous woman’s are Muslim. In terms of residence, 56% of monogamous women and 71.5% of polygamous women live in rural areas, respectively (Table 1).

**Table 1:** Socio-demographic character of Monogamous and Polygamous women in Worebabo Woreda, South Wollo, Ethiopia, 2021

Variable	Category	Monogamous women (N = 352)	Polygamous women (N = 351)	X^2^	*P*-value
		No	No		
Respondents age	15–24	40 (11.4%)	21 (6.05%)	35.59	0
	25–34	166 (47.2%)	106 (30.2%)		
	35–49	146 (41.5%)	224 (63.8%)		
Educational status	No formal education	156 (44.3%)	181 (51.6%)	13.88	0.002
	Primary education	130 (36.9%)	138 (39.3%)		
	Secondary and above	66 (18.8%)	32 (9.1%)		
Occupation	Farmer	167 (47.4%)	186 (53%)	2.76	0.397
	House Wife	109 (31%)	92 (26.2%)		
	Business woman	60 (17%)	60 (17.1%)		
	Government employee	16 (4.5%)	13 (3.7%)		
Religion	Muslim	226 (64.2%)	249 (70.9%)	3.63	0.064
	Orthodox	126 (35.8%)	102 (29%)		
place of residence	Rural	197 (56%)	251 (71.5%)	18.37	0
	Urban	155 (44%)	100 (28.5%)		
Years at first marriage	< 18 years old	175 (49.7%)	207 (59%)	6.07	0.014
	> = 18 years old	177 (50.3%)	144 (41%)		
Husband age	15–24	14 (4%)	0	29.3	0
	25–34	67 (19%)	33 (9.4%)		
	35–49	271 (77%)	318 (90.6%)		
Husband educational status	No formal education	116 (33.0%)	148 (42.2%)	9.48	0.002
	Primary education	151 (42.9%)	146 (41.6%)		
	Secondary and above	85 (24.1%)	57 (16.2%)		
Husband occupation	Farmer	218 (61.9%)	190 (54.1%)	4.8	0.183
	Business man	113 (32.1%)	140 (39.9%)		
	Government employee	21 (6.0%)	21 (6%)		

### Reproductive character

Among all married women, 91.5% of monogamous and 78.3% of polygamous women have a history of pregnancy. Among these women, 22.2% and 21.4% of monogamous and polygamous women had 3–4 pregnancies, respectively. The result also shows that there is a statistically significant difference (X2 = 43.16, *P*-value = 0.001) between the two groups and the number of pregnancies they had. In terms of abortion history, 7.7% and 14.5% of monogamous and polygamous women, respectively, had a history of abortion. With regard to the number of children a respondent has, 18.2% and 34.8% of monogamous and polygamous women have more than or equal to five children, respectively (Table 2).

**Table 2:** Reproductive Character of monogamous and polygamous women in Worebabo Woreda in 2021

Variable	Category	Monogamous women (N = 352)	Polygamous women (N = 351)	X^2^	*P*-value
		No	No		
History of pregnancy	No	30 (8.5%)	76 (21.7%)	23.6	0
	Yes	322 (91.5%)	275 (78.3%)		
Number of pregnancy	01-Feb	197 (61.2%)	106 (38.5%)	43.16	0
	03-Apr	46 (14.3%)	26 (9.5%)		
	> = 5	79 (24.5%)	143 (52%)		
Number of delivery	0	38 (10.8%)	80 (22.8%)	78.07	0.034
	01-Feb	217 (61.6%)	103 (29.3%)		
	03-Apr	31 (8.8%)	74 (21.1%)		
	> = 5	66 (18.8%)	94 (26.8%)		
History of still birth	No	305 (86.6%)	300 (85.5%)	0.2	0.65
	Yes	47 (13.4%)	51 (14.5%)		
History of abortion	0	235 (66.8%)	292 (83.2%)	31.58	0
	1	104 (29.5%)	59 (16.8%)		
	2	13 (3.7%)	0		
Number of live children	0	101 (28.7%)	90 (25.6%)	16.81	0.001
	01-Feb	151 (42.9%)	115 (32.8%)		
	03-Apr	36 (10.2%)	38 (10.8%)		
	> = 5	64 (18.2%)	108 (30.8%)		
women’s desire to next child	Less than two years	114 (32.4%)	99 (28.2%)	1.51	0.367
	After two years	124 (35.2%)	134 (38.2%)		
	No desire	114 (32.4%)	118 (33.6%)		
Respondents husband number of desired children	Same number	112 (31.8%)	44 (12.5%)	106.1	0.554
	More children	112 (31.8%)	241 (68.7%)		
	Fewer children	128 (36.4%)	46 (13.1%)		
	Don’t know	0	20 (5.7%)		

### Knowledge on modern contraception

From the total respondents, 94.8% and 91.5% of monogamous and polygamous women have heard about modern contraceptive, respectively, and for most of the respondents, the source of information was Health extension workers, which is 29.0% and 31.1%, respectively. With regard to the types of modern contraceptive methods, injectables were the most commonly mentioned by 70.7% and 60.7% of monogamous and polygamous women, respectively. Finally, when we compute the knowledge of the respondents based on knowledge related questions, 55.1% and 48.1% of monogamous and polygamous women have good knowledge of modern contraceptive methods (Table 3).

**Table 3:** Knowledge on contraceptive methods of monogamous and polygamous women in Worebabo Woreda in 2021

Variable	Category	Monogamous women (N = 352)	Polygamous women (N = 351)	X^2^	*P*-value
		No	No		
Respondent ever heard about modern contraceptive methods	No	18 (5.1%)	30 (8.5%)	3.25	0.075
	Yes	334 (94.9%)	321 (91.5%)		
Respondent knows where modem contraceptive is provided	No	18 (5.1%)	30 (8.5%)	3.25	0.071
	Yes	334 (94.9%)	321 (91.5%)		
Knows pills (coc, pop)	No	105 (29.8%)	221 (63%)	77.5	0
	Yes	247 (70.2%)	130 (37%)		
Knows injectable	No	103 (29.3%)	138 (39.3%)	7.8	0.005
	Yes	249 (70.7%)	213 (60.7%)		
Knows Implants	No	208 (59.1%)	196 (55.8%)	0.76	0.383
	Yes	144 (40.9%)	155 (44.2%)		
Knows IUCD	No	244 (69.3%)	243 (69.2%)	0.001	0.98
	Yes	108 (30.7%)	108 (30.8%)		
Knows Condom	No	226 (64.2%)	197 (56.1%)	4.78	0.029
	Yes	126 (35.8%)	154 (43.9%)		
Knows surgical method	No	270 (76.7%)	267 (76.1%)	0.039	0.843
	Yes	82 (23.35%)	84 (23.9%)		
Respondent knowledge on modern contraceptive methods	Poor knowledge	158 (44.9%)	182 (51.9%)	3.41	0.065
	Good knowledge	194 (55.1%)	169 (48.1%)		

The qualitative part of the study also found that due to discussions with Health extension workers and health education sessions in health posts and health centers, most key informant mothers had awareness about modern contraceptives and the benefits they yield for women and children’s health.

The key informants stated that “I have heard about family planning at healthcare facility, at home and through conversation with friends.”

### Utilization of modern contraceptive

**Fig. 1:**
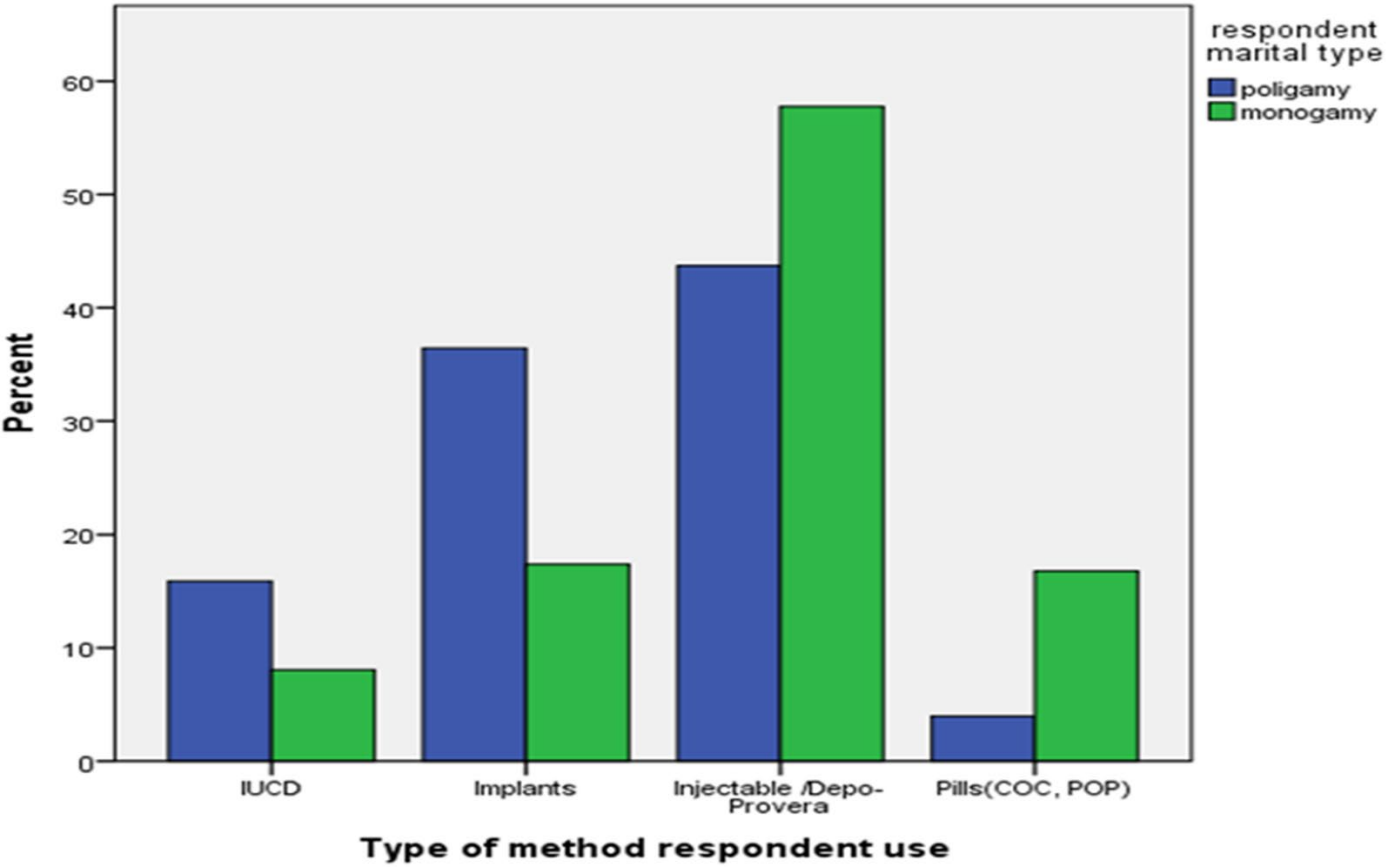
Utilization of modern contraceptive methods by Monogamous and Polygamous married reproductive aged women in Worebabo Woreda in 2021

**Fig. 2:**
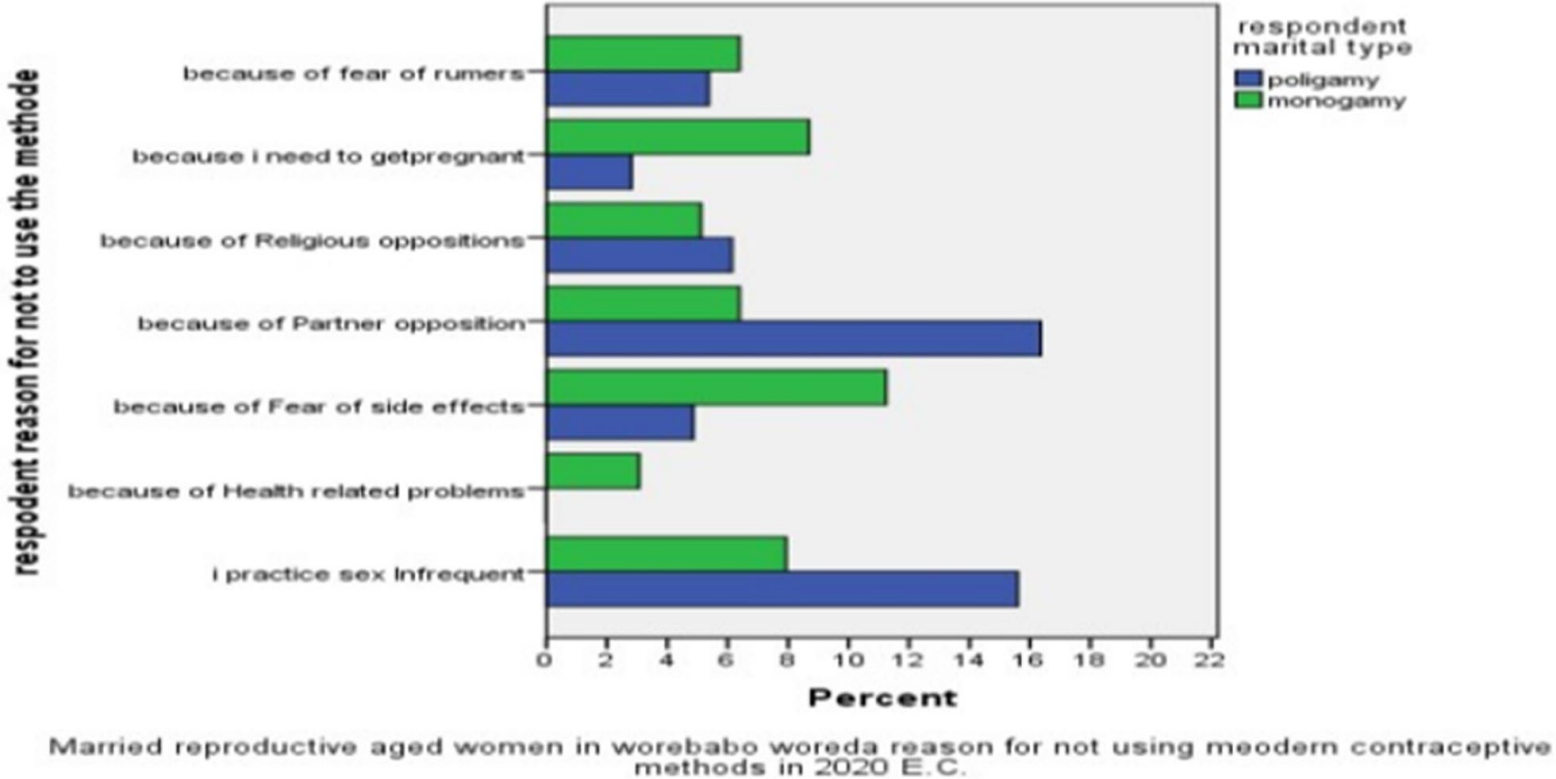
Married reproductive aged women in Worebabo Woreda reasons for not using modern contraceptive methods in 2021

With regard to the current utilization of modern contraceptive methods, 45.7% and 43.0% of Monogamous and polygamous women use modern contraceptive methods. Among the monogamous women who use modern contraceptive methods, 57.7% rely on injectables, 17.4% on implants, 16.8% on pills, and 8.1% on IUCD, while among polygamous women, 43.7% rely on Injectable, 36.4% on Implants, 4.0% on pills, and 15.9% on IUCD ( Fig. 1) Concerning reasons for not using the method 23.5% since they practice sex infrequently, 3.1% because of health-related problems, 16.1% because of fear of side effects, 22.8% because of partner opposition, 11.5% because they need to get pregnant, 11.5% because of fear of rumors 5.7% and 11.3 because of religious opposition are mentioned by monogamous and polygamous women (Fig. 2).

Most of the participants of the key informant interview mention that they prefer to use injectable due to its simplicity to use the method, can be used for a short period of time, no procedure is needed to discontinue and also easy to hide. (Table 4).

“I prefer using injectable since my husband didn’t allow me to use any methods”

**Table 4:** Utilization of modern contraceptive methods by monogamous and polygamous women in Worebabo Woreda in 2021

Variable	Category	Monogamous women (N = 352)	Polygamous women (N = 351)	X^2^	*P*-value
		No	No		
Current use any modern contraceptive	No	191 (54.3%)	200 (57%)	0.526	0.468
	Yes	161 (45.7%)	151 (43%)		
Type of contraceptive method used	Pills (COC, POP)	27 (16.8%)	6 (4%)	29.71	0
	Injectable	93 (57.8%)	66 (43.7%)		
	IUCD	13 (8.1%)	24 (15.9%)		
	Implants	28 (17.4%)	55 (36.4%)		
Duration of contraceptive use	< 1 year	38 (23.6%)	18 (11.9%)	16.73	0.421
	1–2 years	42 (26.1%)	65 (43%)		
	3–5 years	40 (24.8%)	23 (15.2%)		
	6–10 years	20 (12.4%)	24 (15.9%)		
	> 10 years	21 (13.0%)	21 (13.9%)		
Reason to use a method	Limiting birth	70 (43.5%)	68 (45%)	0.076	0.783
	Spacing birth	91 (56.5%)	83 (55%)		
Place where a respondent get a method	Private clinic	37 (23.0%)	24 (15.9%)	19.3	0.046
	District hospital	15 (9.3%)	0		
	Health Center	67 (41.6%)	84 (55.6%)		
	health post	42 (26.1%)	43 (28.5%)		
Getting clear information by Health care provider (HCP)	No	175 (49.7%)	177 (50.4%)	0.036	0.85
	Yes	177 (50.3%)	174 (49.6%)		
Respondent intention to continue using method	No	60 (37.3%)	45 (29.8%)	1.94	0.164
	Yes	101 (62.7%)	106 (70.2%)		
Respondent reason to discontinue using method	Husband disapproval and fear	0	5 (11.1%)	7.9	0.101
	religious opposition	11 (18.3%)	7 (15.6%)		
	Need to get pregnant	34 (56.7%)	26 (57.8%)		
	Fear of Rumor	7 (11.7%)	3 (6.7%)		
	Fear of side effect of methods	8 (13.3%)	4 (8.9%)		
Respondent ever start using method and discontinue	No	238 (67.6%)	226 (64.4%)	0.815	0.367
	Yes	114 (32.4%)	125 (35.6%)		
Respondent reason to discontinue method	Husband disapproval	11 (9.6%)	16 (12.8%)	5.937	0.039
	religious opposition	7 (6.1%)	16 (12.8%)		
	Need to have many children	11 (9.6%)	13 (10.4%)		
	Fear of rumor	34 (29.8)	40 (32%)		
	Fear of side effect of methods	51 (44.7%)	40 (32%)		

Most of the key informant mentions some sociocultural factors as hindrance of modern contraceptive utilization. Myths and misconceptions about modern methods which include exaggerated or inaccurate reports about side effects, misconceptions about short- or long-term health problems and negative stereotypes about persons who practice family planning is mentioned by participants.

“As I heard from my friends and neighbors using modern contraceptive causes infertility, birth defects, cancer, stomach ache and condoms were associated with promiscuity, decreased libido, un healthy weight gain and loss”

“Using modern contraceptive methods such as implants causes painful arms on women especially when they perform their day today activity, made them to need additional food, cause psychosis and headache.”

“They implement the family planning program in order to limit and decrease our population since modern family planning methods cause infertility and other medical problems such as cancer, diabetes, hypertension, anemia, kidney stone and other disease.”

The other socio-cultural factor that mentioned by participants is fear of tags given by husbands and community members to women who use modern contraceptive methods.

“I am afraid to use modern contraceptive methods because if my husband finds out am using he will think that i am cheating on him or will leave him to another man”

“I heard when some people talked behind women who uses modern contraceptive as she was unfaithful to her husband and sleep with others easily because of this e and my friend afraid utilizing methods.”

The other topic mentioned by participants as a hindering factor to utilize modern contraceptive method is its lack of acceptance by culture and religious leaders.

“One day when my husband and I discuss, I asked him to give me permission to use modern contraceptive method but he said that we should decide after consulting our religious leader who told as using modern contraceptive is not allowed since it is an act of violating the will of God.”

Key informant women in polygamous relation mentions that the nature of their marital relationship affects their utilization of modern contraceptive since they are in competitions with co-wives to win their husband.

“I do not want to use modern contraceptive method because I need to have as many children as I can. Having many children will help me to win my husband beside my children will help me when I get older even if my husband stop supporting me.”

### Respondent attitude on modern contraceptive methods

Among all married women interviewed about their attitude towards modern contraception methods 46.9% and 58.1% monogamous and polygamous women perceive that using modern contraceptive methods is important for the wellbeing of children and family. As for either do they haven’t fear of social stigma by the community or not 66.2% and 42.2% of monogamous and polygamous women haven’t fear of social stigma by the community. Among the respondents, 67.6% and 47.0% of monogamous and polygamous women have a family pressure to use of modern contraceptive methods. (Table 5).

**Table 5:** Perception of modern contraceptive methods among monogamous and polygamous women in Worebabo Woreda in 2021

Variable	Category	Monogamous women (N = 352)	Polygamous women (N = 351)	X^2^	*P*-value
		No	No		
Do you think FP is important to the wellbeing children and the family?	No/not sure	187 (53.1%)	147 (41.9%)	8.91	0.003
	Yes	165 (46.9%)	204 (58.1%)		
Do you haven’t fear of social stigma?	No	119 (33.8%)	203 (57.8%)	40.87	0
	Yes	233 (66.2%)	148 (42.2%)		
Do you think religious leaders support FP use?	No	158 (44.9%)	147 (41.9%)	3.99	0.046
	Yes	194 (55.1%)	204 (58.1%)		
Who is responsible to make decisions about number of children in a family?	Husband	127 (36.1%)	132 (37.6%)	33.63	0.011
	Both	168 (47.7%)	105 (29.9%)		
	Respondent	57 (16.2%)	114 (32.5%)		
Don’t you accept myths about contraceptives	No	146 (41.5%)	186 (53%)	9.34	0.002
	Yes	206 (58.5%)	165 (47%)		
Do you have family pressure to use of modern contraceptive methods?	No	114 (32.4%)	186 (53%)	36.48	0
	Yes	238 (67.6%)	165 (47%)		

### Factors associated with modern contraceptive use among married reproductive age women who are in monogamous relationship

Bivariate and multivariate logistic regression models were fitted to determine the presence of an association between the dependent variable and the independent variables at (*P* < 0.05) lev-el of significance. Those variables that had a *P*-value ≤ 0.2 with modern contraceptive utilization in the bivariate analysis were hired for multiple logistic regression analysis.

In the final multi-variable logistic regression model, variables such as educational status [AOR = 2.31, 95% CI (1.231, 4.334)], number of live children [AOR = 2.64, 95% CI (1.248, 5.242)], undesired fertility [AOR = 2.965, 95% CI (1.512,6.357)], getting clear information by Health care provider [AOR = 3.910 (2.253, 7.329)], who decide on the number of children in respond-ents family [AOR = 2.819, 95% CI (1.402, 5.671)], family pressure [AOR = 3.616, 95% CI (1.861, 7.024)], and accept myths [AOR = 1.824, 95% CI (1.017, 3.271)] about contraceptives were significantly associated with the utilization of modern contraceptive methods.

### Factors associated with modern contraceptive use among married reproductive age women who are in polygamous relationship

Bivariate and multivariate logistic regression models were fitted to determine the presence of an association between the dependent variable and the independent variables at (*P* < 0.05) lev-el of significance. Those variables which had a *P*-value ≤ 0.2 with modern contraceptive utilization in the bivariate analysis were hired for multiple logistic regression analysis.

In final multi-variable logistic regression model variables such as educational status [AOR = 2.321, 95% CI (1.298, 4.151)], religion [AOR = 2.382, 95% CI (1.298, 4.390)], undesired fertility [A0R = 2.309, 95% CI (1.125, 4.741)], getting clear information by Health care provider [AOR = 5.414, 95% CI (3.032, 9.669)], who decide on the number of children in respondents family [AOR = 5.216, 95% CI (2.564,10.612)], fear of social stigma [AOR = 3.109, 95% CI (1.697, 5 0.697)], and accept myths about contraceptives [AOR = 1.895, 95% CI (1.072, 3.350)] were significantly associated with utilization of modern contraceptive methods.

### Factors associated with modern contraceptive use among married reproductive age women who are in monogamous and polygamous relationship

Bivariate and multivariate logistic regression models were fitted to determine the presence of an association between the dependent variable and the independent variables at (*P* < 0.05) level of significance. Those variables which had a *P*-value ≤ 0.2 with modern contraceptive utilization in the bivariate analysis were hired for multiple logistic regression analysis.

In final multi-variable logistic regression model variables using such as educational status, religion, undesired fertility, getting clear information by Health care provider, who decide on the number of children in respondents family, fear of social stigma, family pressure, and accept myths about contraceptives were significantly associated with utilization of modern contraceptive methods. (Table 6).

**Table 6:** Factors associated with modern contraceptive use among married reproductive age women who are in monogamous and polygamous relationship in 2021

Variable	Modern contraceptive utilization		COR (95 CI)	AOR (95 CI)
	Non users	Users		
*Educational status of respondent*				
No formal education	221 (65.57)	116 (34.42)	1	1
Primary level education	132 (49.25)	136 (50.74)	1.963 (1.413,2.726)	2.143 (1.428,3.216)***
Secondary level and above	38 (38.77)	60 (61.22)	3.008 (1.891,4.786)	1.843 (1.044,3.253)*
*Type of respondents religion*				
Muslim	273 (60.93)	175 (39.06)	1	1
Orthodox	118 (46.27)	137 (53.72)	1.726 (1.256,2.374)	1.704 ( (1.144,2.539)**
*Desire time for another child*				
Less than two year	149 (69.95)	64 (30.07)	1	1
After two year	148 (50)	148 (50)	1.730 (1.180,2.538)	1.672 (1.06,2.639)*
No desire	94 (50)	94 (50)	3.418 (2.307,5.064)	3.17 (1.939,5.183)***
*Getting clear information by Health care provider (HCP)*				
No	249 (70.73)	103 (29.26)	1	1
Yes	142 (40.45)	209 (59.54)	3.558 (2.601,4.867)	4.624 (3.132,6.828)***
*Who decide on number of children*				
Husband	196 (75.67)	63 (24.32)	1	1
Both	124 (45.42)	149 (54.57)	3.738 (2.581,5.415)	3.054 (1.93,4.832)***
Respondent	71 (41.52)	100 (58.47)	4.382 (2.890,6.643)	4.775 (2.850,8.003)***
*Do you haven’t fear of social stigma?*				
No/Not sure	233 (72.36)	89 (27.63)	1	1
Yes	158 (41.469)	223 (58.53)	3.695 (2.688,5.079)	2.482 (1.666,3.699)***
*Do you have family pressure to use of modern contraceptive methods?*				
No/Not sure	214 (69.70)	93 (30.29)	1	1
Yes	177 (44.69)	219 (55.30)	2.847 (2.080,3.897)	1.855 (1.351,2.75)**
*Don’t you accept myths about contraceptives*				
No	227 (68.37)	105 (31.62)	1	1
Yes	164 (44.20)	207 (55.79)	2.729 (2.003,3.717)	1.878 (1.278,2.761)**

Back ward stepwise multiple logistic regression was used to assess the independent effect of explanatory variables

*P-value < 0.05, **P-value < 0.01, ***P-value < 0.001

## Discussion

The prevalence of women who use modern contraceptives among women who are in monogamous relationships is 161 (45.7%), [95%, CI (40.2, 51.0)], and 151 (43.0%) on polygamous women [95%, CI (37.9, 48.4)].This result shows that there is no difference between the two groups with regard to the utilization of modern contraceptives. These findings was consistent with other similar studies conducted in Ethiopia South nation [27]. On the other hand, studies done in Ethiopia found that polygamous women are less likely to use modern contraceptive methods when compared with monogamous women [3]. On the contrary, a comparative study done in Nigeria showed that contraception was found to be more widely used by women in polygamous than in monogamous marriages [28]. These variations might be due to dissimilarities in socio-cultural, health service utilization, and economic variations among study participants.

Married women knowledge and method preference, Couples discussion and decision on fertility and methods use, Fear of Side effects and misconceptions, Fear of husband and community labeling, not accepted by culture and religious leaders, Competition between co wives in polygamous relation are themes identified from qualitative part of the study.

In accordance with this study finding, the odds of using modern contraceptive methods among married women with primary and secondary level education were 2.14 and 1.84 times higher than married women without formal education, respectively. The relationship between utilization of modern contraceptive, and educational status of respondents was also shown in similar studies done in different places of Ethiopia [21, 29]. Being educated will increase the awareness of contraceptive utilization and its advantages.

The study result showed that respondents’ type of religion has a statistically significant association with the utilization of modern contraceptives, the odds of utilization of modern contraceptive methods is higher for respondents who follow the orthodox Christian religion by 1.704 times than the respondents who follow the Muslim religion. This result is supported by a study done in western Ethiopia [21]. The qualitative study also showed that religious leaders' attitudes toward modern contraceptive methods are negative. Because of this, married women got difficulties from their husbands and others when they try to utilize modern contraceptive methods. Although religion has a major influence on a variety of social attitudes, the relationship between religion and its insight on contraceptives has remained largely unexplored [30].

The odds of using modern contraceptive methods among women who desire to have other child after two years and those who don’t have a desire to have another child are 1.672 and 3.17 times higher than women who desire to have another child within two years, respectively. This finding is in line with a study done in the Debre Birhan District of Ethiopia [13]. It was obvious that women who desired children were not ready to use contraceptives.

The other independent predictor which had a very strong association with married women in monogamous and polygamous relationship was getting clear information by Health care provider. The result showed that the odds of using modern contraceptives by women who have gotten clear information by Health Care providers was found to be 4.62 times higher than those who didn’t got clear information by Health Care providers. This finding is supported by different studies done in different place [31, 32]. This can be explained by Health Care providers give information that covers the advantages and limitations of contraceptive methods, management of common side effects, and how to obtain contraception services which may raise women's overall awareness of different family planning methods.

The study found that the odds of using modern contraceptive methods for women who decide together with their husband and by themselves were 3.0 and 4.7 higher, respectively, than for women whose husband decides on the number of children. This result is in line with the study done in western Ethiopia, the study found that women who made joint decisions about the number of children were more likely to use modern contraceptives than those who did not make joint decisions [29]. The qualitative study also showed that married women’s ability to decide on the number of children they want to have compromised by their husbands, since like in most rural parts of the country, the husband is a source of income, which gives him the power of decision.

The perception of women about fear of social stigma towards modern contraceptive utilization has an independent effect, those who haven’t fear of social stigma have higher odds of using modern contraceptives by 2.48 times than women who have a fear of social stigma towards using modern contraceptive methods. This finding is supported by a study done in Kenya, and India [33, 34]. Hence, those women who have a fear of social stigma for using modern contraceptives are less likely to freely decide and use it.

Women who have family pressure to use of modern contraceptive have higher odds of using modern contraceptives by 1.85 times than women who have not family pressure to use modern contraceptives. This finding is supported by other studies done in Malawi and India [35, 36]. The possible reasons might be the family approval and consent, which motivates the women to make a decision towards modern contraceptive utilization.

In accordance with this study finding, women who didn’t accept myths about contraceptives were found to be 1.87 more likely to use modern contraceptives than those women who accepted myths about contraceptives. This finding is supported by a study done in different places [37, 38]. This could be due to some of the misconceptions including rumors’ about contraceptives, which can reduce the interest of women to use modern contraceptives.

## Strength and limitation of the study

The study was based on data obtained through a cross-sectional examination of the respondents and as such the data was susceptible to response bias. However, its major strength was the mixed methods employed to explore the associated factors of contraceptive use.

## Conclusion

This study identified lower modern contraceptive method utilization by married women in the study area, and the prevalence of utilization between the two groups is not different from one another. It’s recommended that District health office and concerned stakeholders done interventions that scale up contraceptive utilization by refute myths and misguided beliefs about modern contraceptives, and suggestions have been made for mass media and family planning health campaigns to improve awareness about the benefits of modern contraceptives and to change social norms about their use. It is also mandatory for all stockholders to work on increasing women's empowerment by helping women to get education. Training of reproductive health service providers to interact with and respond to incorrect information held by clients is recommended. Finally, awareness creation should be done by health extension professionals about modern contraception to religious leaders, and significant others.

## Data Availability

The datasets generated and analyzed during the current study are not publicly available due to the risks in identifying participants as true anonymization would be difficult to guarantee, but subsets of the data can be available from the corresponding author upon a reasonable request.
